# Re-evaluating Methods for Assessing Differences in Response in Ileal vs Colonic Crohn’s Disease: A Post-hoc Analysis of the FITZROY Trial

**DOI:** 10.1093/ecco-jcc/jjae113

**Published:** 2024-07-20

**Authors:** Christopher Ma, Brian G Feagan, Zhongya Wang, Guangyong Zou, Michelle I Smith, Lisa M Shackelton, Bruce E Sands, Remo Panaccione, Geert R D’Haens, Séverine Vermeire, Vipul Jairath

**Affiliations:** Division of Gastroenterology & Hepatology, University of Calgary, Calgary, AB, Canada; Department of Community Health Sciences, University of Calgary, Calgary, AB, Canada; Medical Research & Development, Alimentiv Inc., London, ON, Canada; Medical Research & Development, Alimentiv Inc., London, ON, Canada; Division of Gastroenterology, Schulich School of Medicine & Dentistry, Western University, London, ON, Canada; Department of Epidemiology & Biostatistics, Western University, London, ON, Canada; Medical Research & Development, Alimentiv Inc., London, ON, Canada; Medical Research & Development, Alimentiv Inc., London, ON, Canada; Department of Epidemiology & Biostatistics, Western University, London, ON, Canada; Medical Research & Development, Alimentiv Inc., London, ON, Canada; Medical Research & Development, Alimentiv Inc., London, ON, Canada; Dr. Henry D. Janowitz Division of Gastroenterology, Icahn School of Medicine at Mount Sinai, New York, NY, USA; Division of Gastroenterology & Hepatology, University of Calgary, Calgary, AB, Canada; Medical Research & Development, Alimentiv Inc., London, ON, Canada; IBD Unit, Division of Gastroenterology, Amsterdam University Medical Centers, Amsterdam, The Netherlands; Department of Gastroenterology and Hepatology, University Hospitals Leuven, Leuven, Belgium; Department of Chronic Diseases & Metabolism, Catholic University Leuven, Leuven, Belgium; Medical Research & Development, Alimentiv Inc., London, ON, Canada; Division of Gastroenterology, Schulich School of Medicine & Dentistry, Western University, London, ON, Canada; Department of Epidemiology & Biostatistics, Western University, London, ON, Canada

**Keywords:** Disease location, endoscopy, Janus kinase inhibitor, mucosal healing, remission, statistical methods

## Abstract

**Background and Aims:**

The ileum is the most commonly affected segment of the gastrointestinal tract in Crohn’s disease [CD]. We aimed to determine whether disease location affects response to filgotinib, a Janus kinase [JAK] inhibitor, in patients with moderately-to-severely active Crohn’s disease [CD] and applying appropriate methods to account for differences in measuring disease activity in the ileum compared with the colon.

**Methods:**

This post-hoc analysis of data from the FITZROY phase 2 trial [NCT02048618] compared changes in the Crohn’s Disease Activity Index [CDAI] and Simple Endoscopic Score for Crohn’s Disease [SES-CD] among patients with ileal-dominant and isolated colonic CD treated with 10 weeks of filgotinib 200 mg daily or placebo. A mixed effects model for repeated measures was used to test whether ileal disease responded differently when compared with colonic disease, by evaluating for effect modification using the interaction term of treatment assignment-by-disease location.

**Results:**

Numerically greater proportions of patients with isolated colonic disease compared to ileal-dominant CD achieved clinical remission [CDAI < 150, 75.9% vs 41.6%] and endoscopic response [SES-CD reduction by 50%, 52.5% vs 15.5%] at Week 10. However, after adjusting for baseline disease activity by disease location and within-patient clustering effects, there was no significant difference in treatment response by disease location [mean difference in ΔCDAI between ileal-dominant vs isolated colonic disease + 9.24 [95% CI: -87.19, +105.67], *p* = 0.85; mean difference in ΔSES-CD -1.93 [95% CI: -7.03, +3.44], *p* = 0.48.

**Conclusions:**

Filgotinib demonstrated similar efficacy in ileal-dominant and isolated colonic CD when controlling for baseline disease activity and clustering effects.

## 1. Introduction

Crohn’s disease [CD] can involve any segment of the gastrointestinal tract; however, the ileum is the most common location, affecting 50–75% of patients.^1^ Different epidemiological patterns, inflammatory cytokine expression, fecal microbiota composition, and long-term prognosis are observed with ileal vs isolated colonic CD.^2^ Patients with ileal CD are at increased risk for stricturing and penetrating complications and have a >2-fold increased risk for CD-related hospitalization and surgery.^3^ Furthermore, this population is 25–40% less likely to achieve clinical remission with medical therapy.^2,4^ In a previous post-hoc analysis, endoscopic healing rates were lower in the ileum compared to the colon after treatment with adalimumab [30.0% and 62.5%], infliximab [36.7% and 52.4%], vedolizumab [18.6% and 29.0%], and ustekinumab [22.7% and 31.3%].^5^ However, these findings are inherently limited due to: [1] a more narrow range of potential Simple Endoscopic Scores for CD [SES-CD] in the ileum compared with the colon; [2] potential baseline imbalances in endoscopic activity based on disease location; and [3] lack of accounting for clustering effects between colonic segments and within individual patients [patients with colonic disease can have multiple segments involved, leading to substantial increases in the colonic SES-CD at baseline vs the ileum].

Given the poor prognosis associated with ileal CD, determining the optimal treatment strategy for these patients is a priority. Janus kinase [JAK] inhibitors are highly effective in moderately-to-severely active ulcerative colitis, and this mechanism of broad-spectrum immunosuppression also shows promise as a therapeutic strategy in CD for both biologic-naive and -exposed populations.^6,7^ Whether JAK inhibition is equally effective in ileal compared with isolated colonic CD remains unclear. Here, we used a mixed effects model for repeated measures [MMRM] that more appropriately accounts for clustered data and adjusts for potential baseline imbalances, to evaluate for effect modification by disease location in a post-hoc analysis of data from a phase 2 trial of filgotinib, an oral JAK-1 inhibitor.

## 2. Methods

### 2.1. Data source

Methods and results from the FITZROY trial [NCT02048618] have been previously published.^7^ Briefly, FITZROY was a phase 2, double-blind, placebo-controlled trial that enrolled 174 patients with moderately-to-severely active CD, defined by a baseline Crohn’s Disease Activity Index [CDAI] 220–450, SES-CD ≥ 7, with at least one ulcer observed endoscopically.^7^ Patients were randomized 3:1 to once daily filgotinib 200 mg or placebo, for 10 weeks. All endoscopies were evaluated by blinded central readers. For this analysis, patients were classified as having ileal-dominant [ileal only or ileocolonic disease] or isolated colonic disease, based on the location of CD identified on the screening colonoscopy.

### 2.2. Statistical methods

Baseline characteristics were summarized using descriptive statistics. In previous studies, estimates of treatment effects in patients with ileal-dominant versus isolated colonic disease have been presented as either: [1] the proportion of patients achieving clinical or endoscopic response; or [2] the mean change in CDAI or SES-CD scores for each disease location. We have presented these measures for comparative purposes [mean change in CDAI and SES-CD, and the proportion of patients achieving CDAI < 150 for clinical remission and 50% reduction in SES-CD for endoscopic response].

However, neither method accounts for baseline imbalances in disease activity nor clustering effects. Therefore, to more appropriately determine if clinical and endoscopic responses differed in patients with ileal-dominant versus isolated colonic CD, we used a likelihood-based MMRM to evaluate the mean change in CDAI and SES-CD at Week 10 compared to baseline and stratified by disease location. We used the original trial population for assessing change in CDAI [*N* = 174], and the population with available matched colonic and ileal segment evaluation at both baseline and follow-up endoscopically to assess change in SES-CD [*N *= 143]. Analysis included fixed categorical effects of treatment assignment [filgotinib versus placebo], disease location, treatment assignment-by-disease location interaction, and continuous baseline SES-CD or CDAI scores. To determine if treatment response is different by ileal-dominant or isolated colonic disease location, we assessed for evidence of treatment-by-disease location effect modification and tested the interaction term, which was considered significant based on least-squares means if the two-sided *p*-value was < 0.05. An unstructured covariance structure was applied to model the within-subject errors.

All analyses were conducted in SAS version 9.4 [SAS Institute, Cary, NC, USA].

## 3. Results

Demographic characteristics of the trial population have been previously published.^7^ Most patients had ileal-dominant [139/174, 79.9%] and 20.1% [35/174] had isolated colonic disease at baseline. The mean [standard deviation] SES-CD at baseline was 14.6 [6.9] and the mean CDAI was 293.4 [± 54.5].

The mean change in the SES-CD and CDAI at Week 10 appeared to favor patients with isolated colonic CD [Table 1] when using traditional analytic approaches. Among patients receiving filgotinib, the mean SES-CD change was -6.3 [± 5.6] in those with isolated colonic disease and -2.3 [± 5.4] in those with ileal-dominant disease. Approximately half (52.2% [12/23]) of patients with isolated colonic CD achieved a 50% reduction in SES-CD, compared with 15.5% [13/84] of patients with ileal-dominant disease. Expressed as a risk difference [Δ36.7%] or as a relative risk [RR] [0.296], it appears that patients with ileal-dominant disease are less likely to achieve endoscopic response. Similar patterns were observed for mean change in CDAI [-152.9 ± 90.1 for isolated colonic and -117.8 ± 101.8 for ileal-dominant CD], and proportion achieving clinical remission (CDAI < 150] [75.9% [22/35] for isolated colonic and 41.6% [42/84] for ileal-dominant CD).

**Table 1:** Endoscopic and clinical response to filgotinib and placebo treatment at Week 10 based on disease location.

Endoscopic outcomes [N = 143]					
	Ileal-dominant disease [*n* = 115]		Isolated colonic disease [*n* = 28]		*p*-value for effect modification by disease location
	Filgotinib [*n *= 84]	Placebo [*n *= 31]	Filgotinib [*n* = 23]	Placebo [*n* = 5]	
Change in SES-CD compared to baseline, mean [SD]	-2.3 [5.4]	-2.5 [6.6]	-6.3 [5.6]	-8.0 [6.0]	0.48
SES-CD reduction > 50% compared to baseline, *n* [%]	13 [15.5]	4 [12.9]	12 [52.2]	3 [60.0]	0.62

Clinical outcomes [*N* = 174]					
	Ileal-dominant disease [*n* = 139]		Isolated colonic disease *[n* = 35]		*p*-value for effect modification by disease location
	Filgotinib [*n* = 101]	Placebo [*n* = 38]	Filgotinib [*n *= 29]	Placebo [*n* = 6]	
Change in CDAI compared with baseline, mean [SD]	-117.8 [101.8]	-89.2 [113.0]	-152.9 [90.1]	-124.8 [91.3]	0.85
CDAI reduction > 100 points compared with baseline, *n* [%]	62 [61.4]	18 [47.4]	22 [75.9]	4 [66.7]	0.90
CDAI < 150 at Week 10, *n* [%]	42 [41.6]	11 [29.0]	22 [75.9]	2 [33.3]	0.14

CDAI, Crohn’s Disease Activity Index; SD, standard deviation; SES-CD, Simple Endoscopic Score Crohn’s Disease.

However, in mixed effects models adjusted for treatment assignment [filgotinib versus placebo], baseline SES-CD, and within-patient clustering, there was no significant difference in treatment effect based on disease location [Table 2]. The mean difference in change in SES-CD between ileal-dominant and isolated colonic disease was -1.93 [95% CI: -7.30, +3.44, *p* = 0.48]. The mean difference in change in CDAI was also not significantly different between ileal-dominant and isolated colonic disease (+9.24 [95% CI: -87.19, +105.67], *p* = 0.85).

**Table 2:** Effect of treatment with filgotinib relative to placebo on SES-CD and CDAI change by disease location.

Effect of filgotinib vs placebo by disease location on SES-CD		
	Estimate [95% CI]	*p*-value
Ileal-dominant	-0.63 [-2.77, +1.51]	0.56
Isolated colonic	+1.31 [-3.67, +6.28]	0.60
Difference in the mean SES-CD change between ileal-dominant and isolated colonic CD	-1.93 [-7.30, +3.44]	0.48^*^
**Effect of filgotinib vs placebo by different disease location on CDAI**		
Ileal-dominant	-29.23 [-67.03, 8.57]	0.13
Isolated colonic	-38.47 [-127.90, 50.97]	0.40
Difference in the mean CDAI change between ileal-dominant and isolated colonic CD	+9.24 [-87.19, +105.67]	0.85^*^

CDAI, Crohn’s Disease Activity Index; CI, confidence interval; SES-CD, Simple Endoscopic Score for Crohn’s Disease.

^*^ *p*-value for test of effect modification by disease location.

## 4. Discussion

It has been postulated that ileal CD is more challenging to treat compared with isolated colonic disease, and that differences in disease location may represent phenotypically separate conditions.^2^ This has been supported by prior post-hoc analyses that show a lower proportion of patients with ileal CD achieve clinical and endoscopic response with medical therapy and are at higher risk for disease-related complications.^5^ In this post-hoc analysis of the FITZROY trial, we aimed to use more appropriate statistical methods to assess whether ileal CD location affects treatment response. Our ‘traditional’ analyses seem to show that the mean change in CDAI/SES-CD and proportional differences [expressed either as risk difference or relative risk] in clinical remission and endoscopic response are numerically higher in patients with isolated colonic CD. However, these ‘traditional’ approaches may not be appropriate, as they do not account for differences in measuring disease activity in the ileum versus the colon, nor do they account for data clustering or baseline disease activity. Rather, when using an MMRM approach, we demonstrated that ileal disease location was not a significant treatment effect modifier of either clinical or endoscopic response in patients receiving filgotinib, after adjusting for these factors.

Although we used high-quality data and appropriate statistical methods to assess effect modification by disease location, our findings should be considered hypothesis-generating, given that they are derived from a phase 2 trial and the size of the subgroup with isolated colonic disease was small. Our methods adjust for baseline imbalances and within-patient clustering, but we recognize that inherent limitations of the SES-CD, particularly for evaluating ileal CD, remain. The SES-CD does not assess ulcer depth, which may be an important confounder of overall treatment response. For the ileum, scoring of both affected and ulcerated surface area is dependent on the depth of intubation, which may vary between clinical trial sites and within individual patients between visits. Now, a requirement for ≥ 10 cm of terminal ileum intubation is typically mandated for clinical trials to standardize subsequent scoring.^8^

Another hypothesis that should be further evaluated based on our findings is whether JAK inhibition has greater mechanistic consequences and, therefore, may be a preferred therapeutic strategy for patients with ileal CD. The pathophysiology of ileal CD appears to be unique and is characterized by decreased expression of α_4_β_7_ integrin-expressing T lymphocytes, and impaired autophagy with reduced bacterial clearance that activates Th17-mediated inflammation.^9,10^ These differences may reduce treatment efficacy in the ileum with highly specific biologic agents that target individual pathways. In contrast, the ability of JAK inhibitors to modify multiple downstream effector cytokines, including IL-23, IL-22, IL-6, interferon-γ, and tumor necrosis factor, may be associated with better treatment responses in the ileum.^11–14^ It should be noted that these effects may differ among JAK inhibitors. For example, although upadacitinib is effective for moderately-to-severely active CD, phase 2 trials of tofacitinib did not demonstrate a benefit over placebo for achieving clinical remission.^15,16^ In the recently completed phase 3 DIVERSITY trial enrolling patients with moderately-to-severely active CD [NCT02914561], neither filgotinib 100 mg nor 200 mg daily met the coprimary endpoints of clinical remission and endoscopic response at Week 10. However, filgotinib 200 mg daily was significantly better than placebo for maintaining Week 58 clinical remission [43.8% vs 26.4%, *p* = 0.04] and endoscopic response [30.4% vs 9.4%, *p* = 0.004].

Several limitations of our study should be acknowledged. First, there were insufficient patients with purely isolated ileal disease activity for valid comparisons. However, other authors have suggested that patients with ileal-only and ileocolonic disease should be considered similarly, given the involvement of the small bowel.^2^ Second, we recognize that endoscopic healing may not occur within 10 weeks of treatment. However, FITZROY was a phase 2 study without Week 52 endoscopic endpoints for analysis. Whether healing differs in the ileum versus colon long-term may be better assessed in several recent treat-through, 52-week, phase 3, registrational CD trials, including the mirikizumab VIVID and guselkumab GALAXI studies.

## Conclusion

In summary, this post-hoc analysis of the phase 2 FITZROY trial did not demonstrate a differential treatment effect of filgotinib 200 mg daily based on CD location in the ileum vs colon. Analyses using similar methods to adjust for baseline imbalances in activity by disease location and within-patient clustering effects should be undertaken for other CD therapies to confirm our findings.

## Data Availability

All data for this trial were made available by Gilead Sciences, Inc. All analyses, interpretation, and manuscript drafting were performed independently by the study authors. The data that support the findings of this study must be requested from Gilead Sciences, Inc.
